# To determine the role of TRIT1 in the diagnosis, prognosis and immunoinvasion of liver hepatocellular carcinoma

**DOI:** 10.3389/fimmu.2025.1563442

**Published:** 2025-05-05

**Authors:** Xinyu Niu, Xiaona Pan, Guifang He, Chao Xuan, Qingwu Tian, Yuan Yuan, Jingqiu Chen, Yaqi Song, Yujuan Tang, Tingting Zhou

**Affiliations:** ^1^ Department of Clinical Laboratory, The Affiliated Hospital of Qingdao University, Qingdao, Shandong, China; ^2^ Qingdao University, Qingdao, Shandong, China; ^3^ Department of Rehabilitation Medicine, The Affiliated Hospital of Qingdao University, Qingdao, Shandong, China; ^4^ Medical Animal Laboratory, The Affiliated Hospital of Qingdao University, Qingdao, Shandong, China; ^5^ Department of Surgery, Hubei Provincial Hospital of Traditional Chinese Medicine, Wuhan, Hubei, China; ^6^ Hubei Provincial Hospital of Traditional Chinese Medicine, Affiliated Hospital of Hubei University of Chinese Medicine, Wuhan, Hubei, China

**Keywords:** liver hepatocellular carcinoma, tRNA isopentenyltransferase 1, prognosis, immune cells, therapeutic target

## Abstract

**Background:**

TRIT1 is identified as a potential tumor suppressor gene that may be involved in tumor development. Existing research indicates that TRIT1 is significant in the development of certain cancers. However, its specific role in liver cancer remains elusive.

**Methods:**

Expression profiles and clinical data of liver hepatocellular carcinoma (LIHC) patients were retrieved from The Cancer Genome Atlas (TCGA) database. The TRIT1 gene levels between LIHC tissues and normal tissues were compared using the Wilcoxon rank-sum test. Additionally, TRIT1 expression levels were further examined via reverse transcription quantitative polymerase chain reaction (RT-qPCR). Functional enrichment analysis was performed to elucidate the biological pathways associated with TRIT1. Immune cell infiltration patterns were evaluated using single-sample gene set enrichment analysis (ssGSEA). The methylation status of the TRIT1 gene were analyzed using the UALCAN and MethSurv databases. Cox regression analysis and Kaplan-Meier (KM) methods were employed to determine the prognostic value of TRIT1. To create a practical tool for predicting overall survival over time, a nomogram was constructed.

**Results:**

The analysis revealed that TRIT1 expression is significantly higher in LIHC tissues compared to normal tissues. Furthermore, elevated TRIT1 levels were found to be associated with specific subtypes of LIHC, including T3 and stage III. Importantly, TRIT1 overexpression was identified as a negative prognostic marker for overall survival in LIHC patients. Additionally, hypermethylation of the TRIT1 gene was associated with poor prognosis. Moreover, this study demonstrated that high TRIT1 levels were correlated with reduced levels of cytotoxic immune cells in the tumor microenvironment, including B cells, cytotoxic cells, and plasmacytoid dendritic cells (pDCs).

**Conclusions:**

This study provides the first evidence that the presence of TRIT1 can serve as a reliable marker for diagnosis and prognostication of hepatocellular carcinoma. Moreover, TRIT1 emerges as a critical indicator of the potential for cancer infiltration and invasion of the immune system, holding significant implications for the development of targeted therapies for hepatocellular carcinoma.

## Introduction

Liver invasive cancer (LIC), with liver hepatocellular carcinoma (LIHC) being a particularly prominent form, represents a formidable global health challenge, accounting for a substantial proportion of cancer-related mortality. As highlighted by the Global Cancer Statistics 2020 report, LIHC contributed to approximately 905,700 new cases and 830,200 deaths worldwide in 2020 alone, which strongly emphasizes its substantial impact on global cancer - related fatality rates (1). The incidence of liver cancer in China is disproportionately high compared to other countries, with the nation harboring roughly 19% of the world’s population yet accounting for over 50% of all newly diagnosed liver cancer cases and deaths in 2012 (2). However, in the last decade, the mortality and disease burden of liver cancer in China have decreased year by year. It is worth noting that the mortality and disease burden are higher among men and in the rural population, and there are significant disparities across the eastern, central, and western regions of China., with higher mortality and disease burden in men and rural population, and significant differences in eastern, central and western regions of China (3). The high incidence rate of liver cancer has spurred extensive research into the etiology, progression, and treatment strategies for LIC. Despite notable advancements in diagnosis and treatment over the past few decades, the prognosis for LIC patients remains dismal, necessitating novel diagnostic and therapeutic approaches.

The pathogenesis of LIHC is intricate, involving alterations in multiple genes and signaling pathways. Recent research has increasingly implicated genetic polymorphisms and aberrant expression of tumor suppressor genes in the development and progression of LIHC. Among these, tRNA isopentenyl transferase 1 (TRIT1) has emerged as a potential crucial factor in cancer development. TRIT1 is responsible for transferring an isopentenyl group to the A37 position of tRNA molecules, a modification that impacts tRNA stability and translational efficiency (4). Furthermore, TRIT1 has been identified as a candidate tumor suppressor gene, with its downregulation observed in various types of cancer and implicated in tumor development and progression (5).

While existing studies suggest that TRIT1 may play a role in cancer development, its specific contribution to LIHC remains unclear. Therefore, the present study aims to investigate the role of TRIT1 in the diagnosis, prognosis, and immune infiltration in LIHC. Through this research, we aim to not only elucidate the mechanistic role of TRIT1 in LIHC pathogenesis but also to provide novel insights and potential therapeutic targets for the diagnosis and treatment of liver cancer.

## Materials and methods

### Cell culture

Human hepatocellular carcinoma cell lines from Procell (Wuhan, China), including SMCC-7721,LM3, HEPG2, and human hepatic stellate cells LX-2, were cultured in RPMI-DMEM (Gibco,USA). Additionally, the culture medium was supplemented with 1% penicillin-streptomycin solution from Beyotime in Shanghai and 10% fetal bovine serum from SIGMA. All the aforementioned cells were cultured in a controlled environment at 37°C with an atmosphere containing 5% CO_2_.

### Real-time quantitative polymerase chain reaction

Real-time quantitative polymerase chain reaction (qPCR) protocol involved the following steps: Firstly, total RNA was extracted from cells using the RNeasy Mini Kit (Qiagen, Hilden, Germany). Subsequently, cDNA was synthesized utilizing the iScript cDNA Synthesis Kit (Bio-Rad Laboratories, CA, USA). For the qPCR analyses, Takara SYBR Premix Ex Taq (Tli RNaseH Plus, Japan) was employed, and the reactions were performed on an FTC-3000P real-time qPCR system (Funglyn Biotech, Toronto, Canada). The thermocycling conditions were set as follows: an initial denaturation at 94°C for 4 minutes, followed by 40 cycles of 94°C for 20 seconds, 60°C for 10 seconds, and 72°C for 20 seconds. Primer sequences used in the experiments are listed in **Table 1**. GAPDH was used as the normalization control. The relative expression levels of the genes were quantified using the 2-ΔΔCt method.

**Table 1:** Sequences of primers for qPCR sequence.

qPCR sequences	Sequences (5′-3′)
GAPDH (Forward)	TGACTTCAACAGCGACACCCA
GAPDH (Reverse)	CACCCTGTTGCTGTAGCCAAA
TRIT1 (Forward)	CAACGGACCCTACCTCTTGT
TRIT1 (Reverse)	CTCTGCTCTTGGGCAGAAAC

### Data collection from TCGA database

The mRNA expression data and clinical information of LIHC patients used in this study were obtained from two primary databases: The Cancer Genome Atlas (TCGA) and the Genotype-Tissue Expression (GTEx) database. The TCGA database, a product of a multi-institutional collaboration, houses extensive molecular data derived from tumor samples. In contrast, the GTEx database offers genomic and expression data from healthy tissues. Leveraging these resources, the study secured a comprehensive and representative dataset, incorporating both cancerous and normal tissue samples. This facilitated an in-depth analysis of mRNA expression patterns in LIHC patients and explored their association with clinical outcomes.

### Differential expression analysis of TRIT1

Based on the minimum P-value of TRIT1 expression, the researchers divided the cancer TCGA patients into two categories: the TRIT1 high expression group and the TRIT1 low expression group. To further explore the gene expression disparities between these two groups, the researchers applied the R package DESeq2, which was developed by Love et al. in 2014 (6). They established the criteria for differentially expressed genes (DEGs) as an adjusted p-value < 0.05 and |log2-fold change (FC)| > 1. Moreover, the researchers sought to assess the correlation between the expression of the top 10 DEGs and TRIT1. For this purpose, they implemented Spearman correlation analysis, a statistical technique used to quantify the strength and direction of the monotonic relationship between two variables.

### Enrichment analysis

Gene Set Enrichment Analysis (GSEA) is a computational technique used to normalize RNA Seq data sourced from The Cancer Genome Atlas (TCGA). This analytical tool, accessible via the MSigDB platform, facilitates exploration of the biological functions of TRIT1. To elucidate potential biological roles, Gene Ontology (GO) classification and Kyoto Encyclopedia of Genes and Genomes (KEGG) pathway enrichment analyses were performed. These processes are carried out using a gene clustering algorithm implemented in the R programming environment. GO categories are divided into three domains: Biological Process (BP), Molecular Function (MF), and Cellular Component (CC). The analysis of TRIT1-related genes facilitates the identification of enriched biological processes, molecular activities, and cellular components associated with this genetic entity. Furthermore, KEGG pathway enrichment analysis enables investigation of the pathways potentially implicated by TRIT1. The KEGG pathway database documents a wide range of biological pathways, enabling the exploration of molecular interactions and signaling mechanisms connected to specific genes or gene clusters. To ensure the consistency of enrichment results, two criteria must be met: a False Discovery Rate (FDR) below 0.05 and a nominal p-value under 0.05. These criteria enhance the reliability of the findings by minimizing false-positive associations, thereby strengthening confidence in the identified biological functions and pathways.

### Analysis of immune cell infiltration

To assess the level of immune infiltration, 24 immune cell types were included in the analysis. The relative enrichment of these immune cells in LIHC was quantified using single-sample Gene Set Enrichment Analysis (ssGSEA), a method implemented through the R package GSVA (7). Moreover, the correlation between TRIT1 expression levels and the abundance of these 24 immune cell types was explored via Spearman correlation analysis. The Wilcoxon rank-sum test was applied to compare the differences in immune infiltration levels between the high and low TRIT1 expression groups.

### DNA methylation analysis

To explore the potential mechanism underpinning the role of TRIT1 in the development of LIHC, the DNA methylation profile of TRIT1 was analyzed using the UALCAN database (8). This database facilitates an systematic analysis of cancer transcriptome data, with particular focus on the methylation status of the TRIT1 DNA sequence. This region is crucial in governing the expression of the TRIT1 gene. Moreover, to evaluate the clinical relevance of TRIT1 methylation levels, we made use of the MethSurv database (9). This online platform is an indispensable tool for executing multivariate survival analyses based on DNA methylation profiles. Clinical outcome data were integrated with TRIT1 methylation profiles to evaluate its potential as a prognostic indicator in cancer patients. These investigations aimed to characterize the role of TRIT1 methylation in cancer progression. The findings may inform the development of targeted therapeutic approaches and support personalized treatment strategies in clinical oncology.

### Validation of the nomogram

To predict overall survival probability, multivariate Cox analysis was employed to evaluate independent prognostic factors for overall survival. The identified significant predictors were subsequently presented in bar chart. This chart visually represents the relationship between these factors and survival probability. The predictive accuracy of the survival model was evaluated through two complementary approaches. First, calibration analysis was performed by comparing observed versus predicted survival probabilities using calibration plots. Second, model discrimination was quantified using the concordance index (C-index), which measures the agreement between predicted and observed survival outcomes. The bar and calibration charts were created using the R software package known as RMS, which facilitated the visualization of the relationship between independent prognostic factors and overall survival probability. Time-dependent predictive performance was further evaluated through time-dependent receiver operating characteristic (ROC) curve analysis implemented using the temporal ROC software package, which enables the construction of time-dependent receiver operating characteristic (ROC) curves. This approach provides a comprehensive evaluation of predictive performance over time. The analysis of the time-dependent ROC curves permits determination of prediction accuracy at specified time intervals, facilitating evaluation of model reliability in forecasting survival probabilities across different disease stages. This analysis aids in evaluating the reliability and applicability of the predictions in real-world clinical scenarios.

### Survival analysis

To analyze the survival data, the Kaplan-Meier method and log-rank test were applied. The cutoff value for the analysis was determined using the minimum P-value of TRIT1 expression, a gene of interest. Univariate and multivariate Cox regression analyses were conducted to evaluate the influence of clinical variables on patient outcomes. In the univariate analysis, a prognostic variable with a p-value < 0.1 was identified. This variable was then included in the multivariate Cox analysis. Forest plots were generated using the R package ggplot2 to visually represent the results of the multivariate Cox regression analysis.

### Statistical analysis

Data analysis was conducted using R Studio software. The Wilcoxon signed-rank test was applied to analyze the expression differences between LIHC and normal tissues. Comparisons between two groups were performed using one-way analysis of variance. A P-value <0.05 was considered statistically significant.

## Results

### Correlation analysis between TRIT1 and LIHC

**Figure 1:**
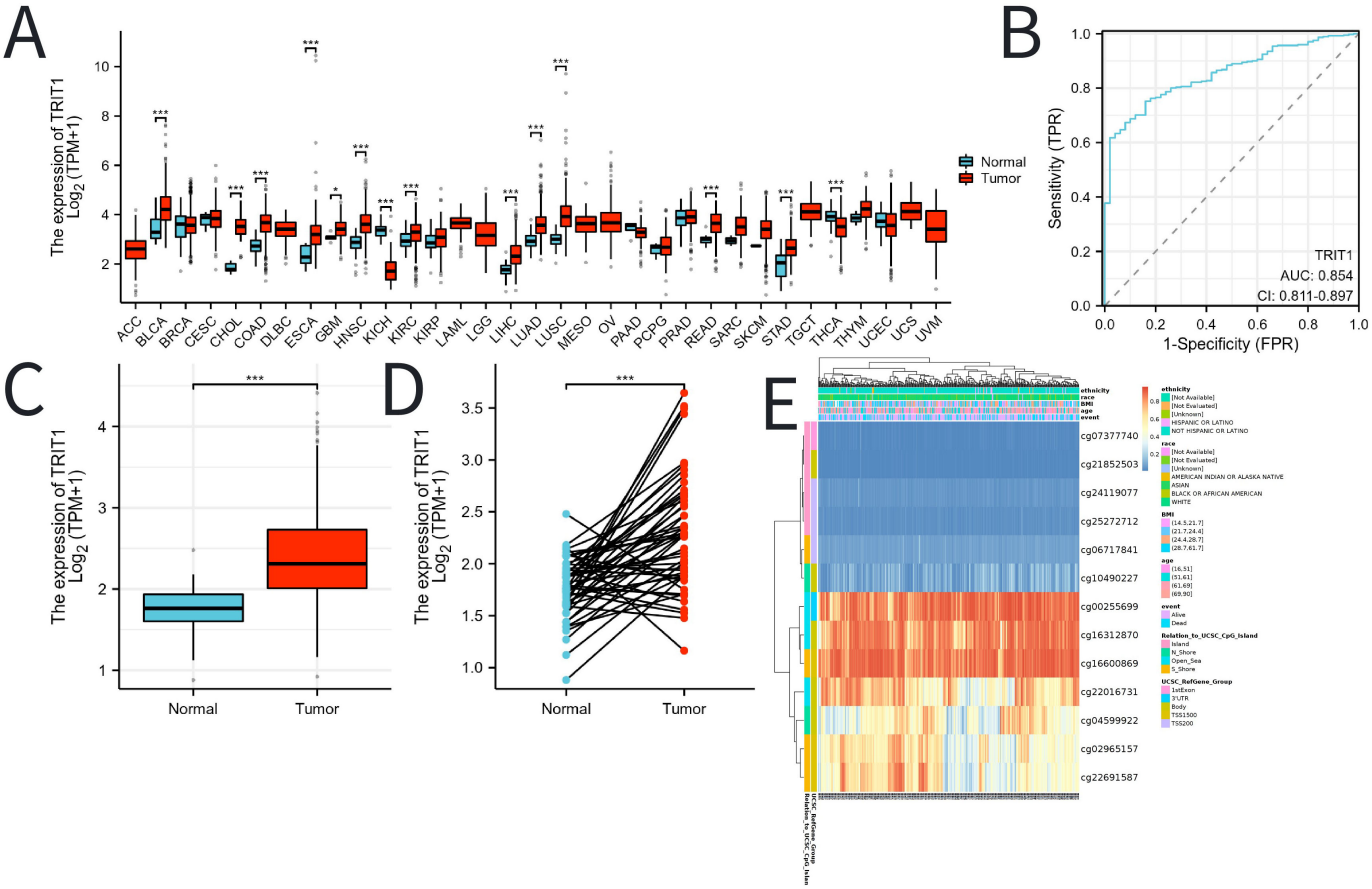
The expression of TRIT1 in tumor and the methylation status of TRIT1 DNA sequence methylation site in LIHC. **(A)** TRIT1 was highly expressed in many solid tumors, including LIHC **(B, C)**. The ROC curve area was 0.854 **(D)**, indicating TRIT1 was a biomarker for diagnostic of LIHC. P-values were calculated with two-tailed unpaired Student’s t-test, *p < 0.05, **p < 0.01, ***p < 0.001. **(E)** Correlation between TRIT1 mRNA expression level and methylation level.

The pan-cancer analysis results indicate that the expression level of TRIT1 is generally higher in various tumors such as esophageal cancer, gastric adenocarcinoma and rectal cancer than in normal tissues (**Figure 1A**). In LIHC, the expression level of TRIT1 is significantly higher than that in normal tissues (**Figure 1B**). Moreover, in 100 LIHC tissues, its expression level is significantly higher than that in paired adjacent tissues (**Figure 1C**). Furthermore, receiver operating characteristic (ROC) curve analysis revealed strong discriminative capacity of TRIT1 expression for LIHC detection, with an area under the curve (AUC) value of 0.854 (**Figure 1D**). These data demonstrate that TRIT1 overexpression is significantly associated with LIHC diagnosis (AUC=0.854), supporting its utility as a diagnostic biomarker. with implications for early detection and effective treatment of LIHC. To elucidate the potential mechanisms underlying TRIT1 overexpression in LIHC, bioinformatic analysis was performed to explore the correlation between TRIT1 expression levels and methylation status. The results identified hypermethylation at specific methylation sites within the TRIT1 genomic locus in LIHC (**Figure 1E**).

**Figure 2:**
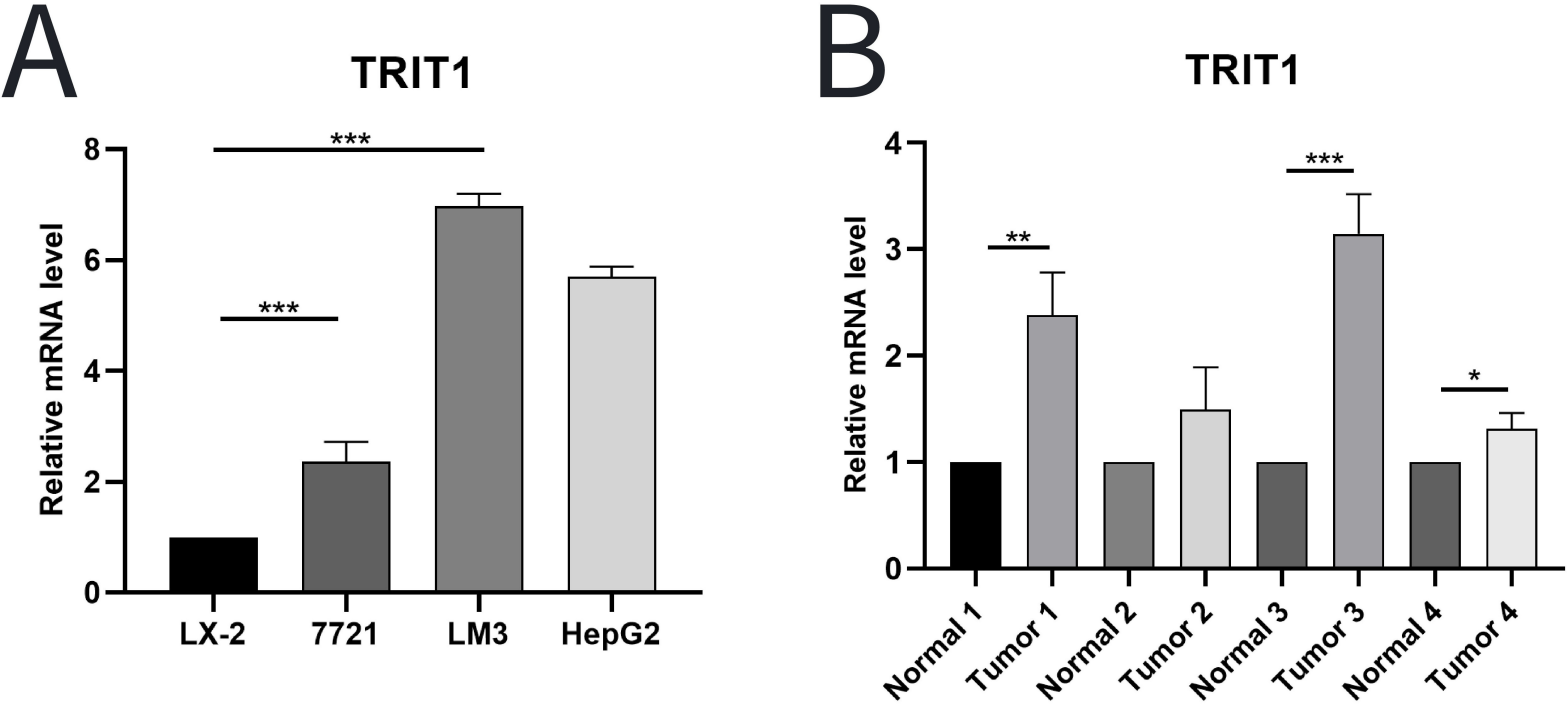
qPCR was used to detect the level of TRIT1 in LIHC. **(A)** Results showed that the expression of TRIT1 in liver cancer cells was higher than that in normal human hepatic stellate cells. **(B)** Results revealed that the level of TRIT1 in liver cancer tissues was higher than that in paracancerous tissues. The experiments were repeated 3 times. Data are shown as means ± SD. P-values were calculated with two-tailed unpaired Student’s t-test, *p < 0.05, **p < 0.01, ***p < 0.001.

A comprehensive analysis using experimental techniques to assess the level and significance of TRIT1 in liver cancer tissues were performed. Initial experiments involved quantitative PCR (qPCR) analysis comparing TRIT1 expression levels between established human hepatocellular carcinoma cell lines and normal human hepatic stellate cells. The results showed that TRIT1 expression was significantly upregulated in liver cancer cell lines compared to normal cell lines (**Figure 2A**). To further validate this finding, the analysis was extended to include actual liver cancer tissue samples taken from patients. Three liver cancer tissues and their adjacent normal tissues were randomly selected for qPCR detection. The experimental results demonstrated consistent overexpression patterns with initial observations, and qPCR data confirmed that the expression level of TRIT1 in liver cancer tissues was significantly increased compared with neighboring normal tissues (**Figure 2B**).

**Figure 3:**
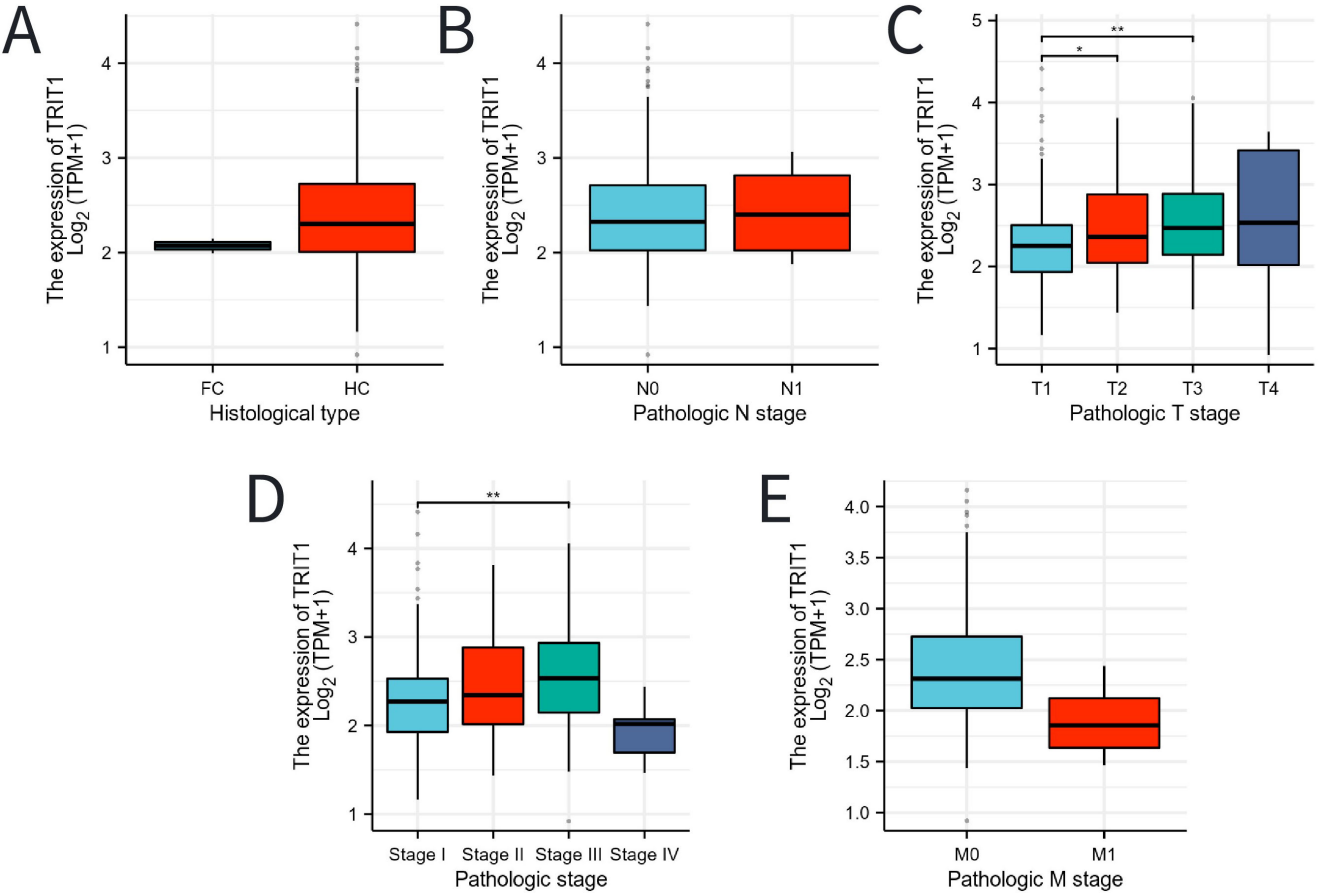
Associations between TRIT1 expression and clinicopathological characteristics. Data were shown for **(A)** histological type, **(B)** N stage, **(C)** T stage, **(D)** pathological stage, **(E)** M stage. P-values were calculated with two-tailed unpaired Student’s t-test, *p < 0.05, **p < 0.01, ***p < 0.001.

The expression level of TRIT1 in hepatocellular carcinoma is higher than that in fibrolamellar carcinoma (**Figure 3A**). Additionally, high TRIT1 expression was not found to be significantly associated with pathological staging, especially between N0 and N1 stages (**Figure 3B**). However, an elevated TRIT1 expression level was observed in the comparison between T3 and T1 stages (**Figure 3C**), and a significant correlation was also found between Stage III and Stage I (**Figure 3D**). Nevertheless, there was no significant relationship between the M stage and TRIT1 expression (**Figure 3E**).

### Functional enrichment analysis of differentially expressed genes related to TRIT1

**Figure 4:**
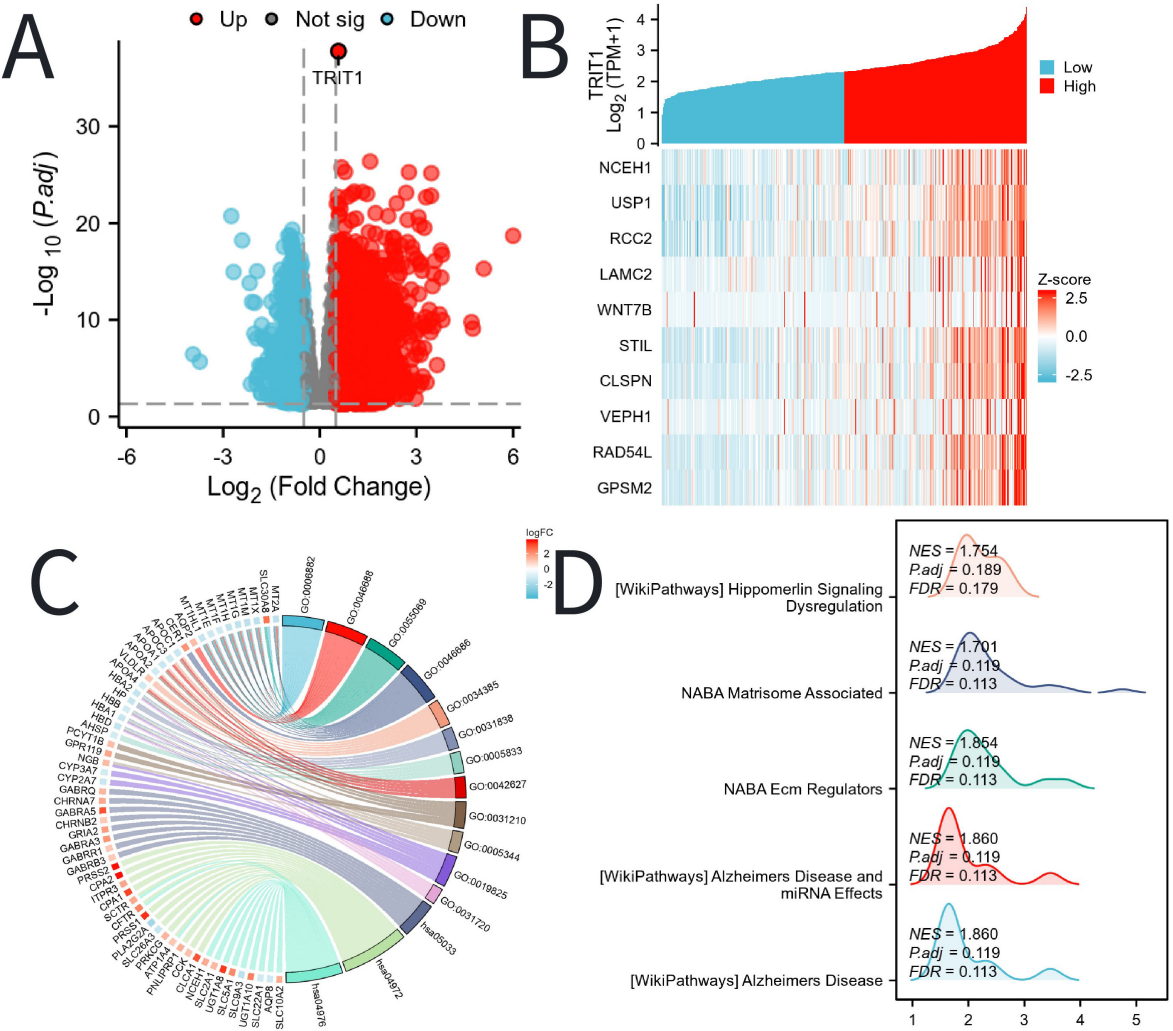
DEGs related to TRIT1 and its functional enrichment analysis utilizing GSEA, GO and KEGG. **(A)** Blue and red dots indicated the vitally down- and up-regulated DEGs in the Volcano plot, respectively. **(B)** The top ten DEGs positively correlated with TRIT1 level. **(C)** KEGG, GO and GSEA **(D)** analysis of DEGs.

TRIT1 has been identified as a significantly highly expressed differentially expressed gene, shown in **Figure 4A**. The study then delves into the top ten genes that interact with TRIT1, namely NCEH1, USP1, RCC2, LAMC2, WNT7B, STIL, CLSPN, VEPH1, RAD54L, and GPSM2. **Figure 4B** illustrates their correlation with TRIT1. The GO and KEGG enrichment analyses were conducted on all DEGs, revealing that the Biological Process (BP) is predominantly enriched in intracellular zinc ion homeostasis, as well as responses to copper and cadmium ions. Cellular Component (CC) is primarily enriched in haptoglobin-hemoglobin complexes, hemoglobin complexes, triglyceride-rich plasma lipoprotein particles, and chylomicrons. Molecular Function (MF) is enriched in oxygen binding, haptoglobin binding, phosphatidylcholine binding, and oxygen carrier activities. Furthermore, the KEGG pathway analysis highlights nicotine addiction, pancreatic secretion, and bile secretion as the most significantly enriched pathways (**Figure 4C**, **Supplementary Table S1**). **Supplementary Table S1** covers all the GO and KEGG enrichment pathways of the DEGs of TRIT1 that appear in **Figure 4C**, and offers a specific description for each of them. Additionally, GSEA subsequently demonstrates that Alzheimer’s Disease biological processes are enriched in the group with high TRIT1 expression (**Figure 4D**).

### The relationship between TRIT1 and prognosis

**Figure 5:**
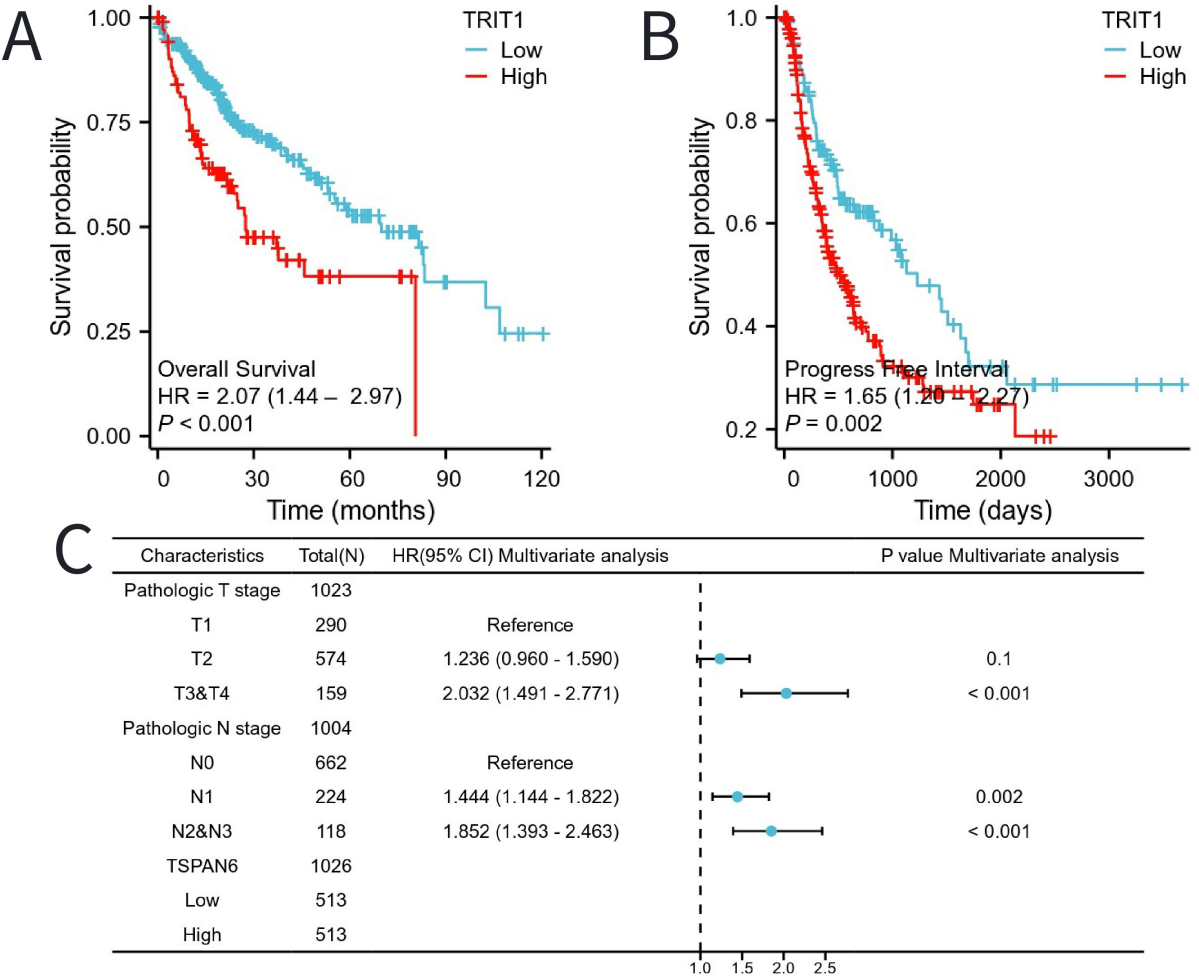
The impact of TRIT1 level on prognosis in LIHC patients was evaluated utilizing Kaplan Meier. **(A)** OS and **(B)** PFI for TRIT patients with high- vs low- TRIT1. **(C)** Forest map of OS with LIHC patients based on multivariate Cox analysis.

The Kaplan-Meier (KM) method was utilized to assess the correlation between TRIT1 levels and the prognosis of liver hepatocellular carcinoma (LIHC). Patients were categorized based on TRIT1 expression, with the cutoff point determined by the expression associated with the smallest p-value. This resulted in two patient groups: one with high-level and another with low-level TRIT1 expression. The comparison demonstrated that patients with high TRIT1 expression had a significantly poorer overall survival (OS) prognosis compared to those with low expression (**Figure 5A**). Additionally, the progression-free interval (PFI) in the high TRIT1 expression group was observed to be shorter (**Figure 5B**). To gain a comprehensive understanding of prognostic indicators in LIHC patients, univariate and multivariate Cox regression analyses were conducted. Certain clinical features were identified as influencing factors for OS, including N1, N2, and N3 stages, as well as T3 and T4 stages (**Figure 5C**). The results of the multivariate Cox regression analysis revealed a P-value of 0.1 for the T2 stage, indicating that, after considering other clinicopathological features, its association with overall survival was not statistically significant at a significance level of 0.05. However, the hazard ratio (HR) for the T2 stage was close to 1, with a narrow 95% confidence interval, suggesting a potential, albeit clinically insignificant, association between T2 stage and survival outcomes. Further research is warranted to confirm this finding’s clinical significance.

**Figure 6:**
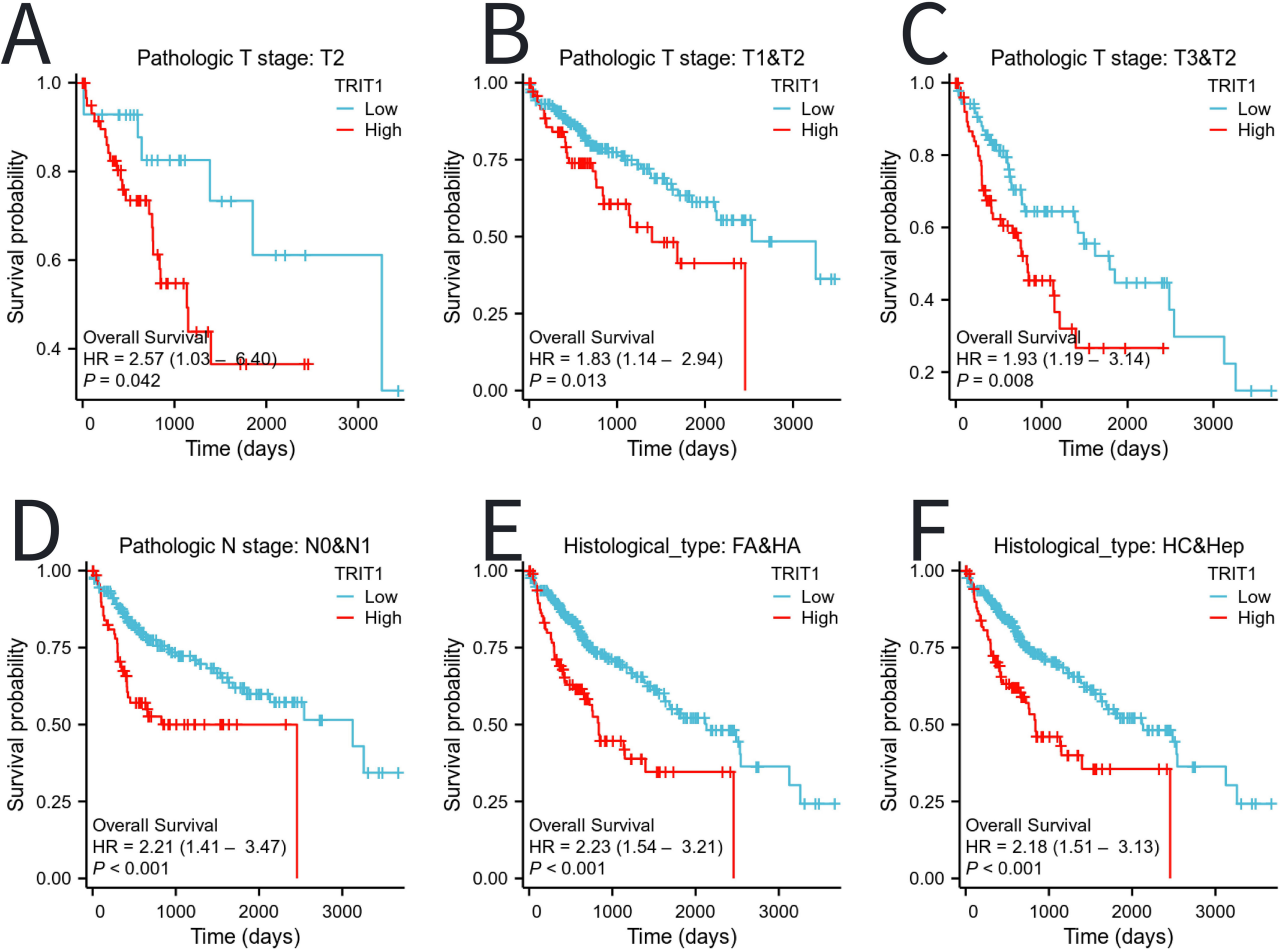
The impact of TRIT1 level on different subgroups prognosis of patients with LIHC discussed by the Kaplan-Meier. **(A–F)** OS survival curves of T2, T1 and T2, T3 and T2,N0 and N1,FA and HA between high- and Low- TRIT1 patients with LIHC.

Moreover, patients with high expression of TRIT1 had poor prognosis in different tumor stages (especially T2, T1&T2, T3&T2), presence or absence of lymph node metastasis (N0&N1), FA&HA, and HC&Hep subgroups (**Figure 6**).

### The relationship between TRIT1 and immunoinvasion

**Figure 7:**
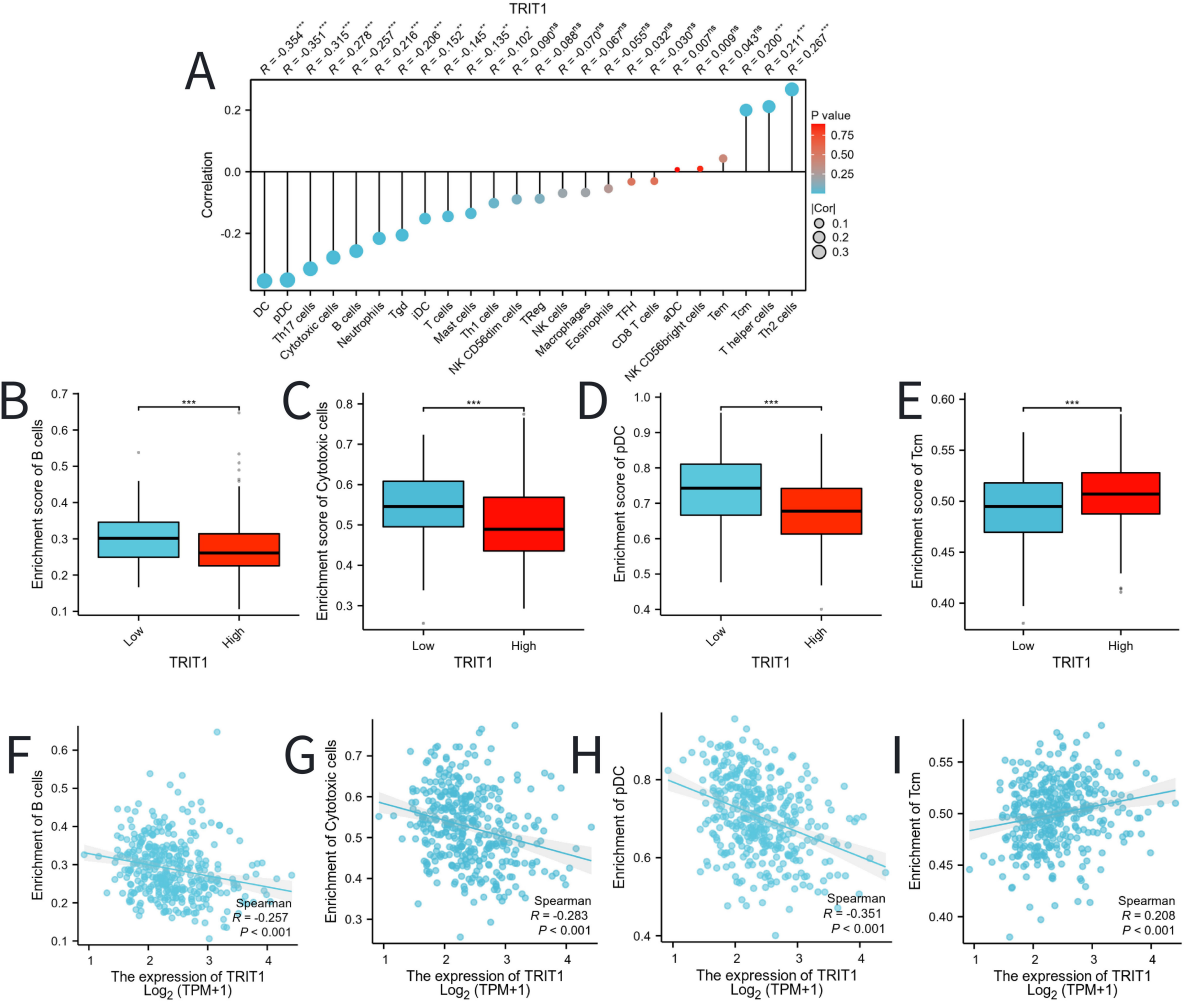
Correlation between TRIT1 level and immune cells infiltration in LIHC. **(A)** Correlation between TRIT1 expression and 24 types of immune cells. **(B–E)** Comparison of immune infiltration levels of immune cells (including pDC、Cytotoxic cells 、and B cells) between high and low TRIT1 level groups. **(F–I)** The expression of TRIT1 was negatively correlated with the level of infiltrating immune cells, including pDC, Cytotoxic cells, Tcm and B cells. P-values were calculated with two-tailed unpaired Student’s t-test, *p < 0.05, **p < 0.01, ***p < 0.001. ns, not significant.

The analysis demonstrated a significant negative correlation between TRIT1 levels and the infiltration of immune cells, particularly impacting pDCs, Cytotoxic cells, and B cells (**Figure 7A**). Furthermore, the figures clearly show that the enrichment scores for B cells, Cytotoxic cells, and pDCs are notably lower in the high TRIT1 expression group compared to the low TRIT1 expression group (**Figures 7B-H**). Conversely, the enrichment score for Tcm cells is notably higher (**Figure 7I**).

### Construction and validation of a nomogram based on the independent factors

**Figure 8:**
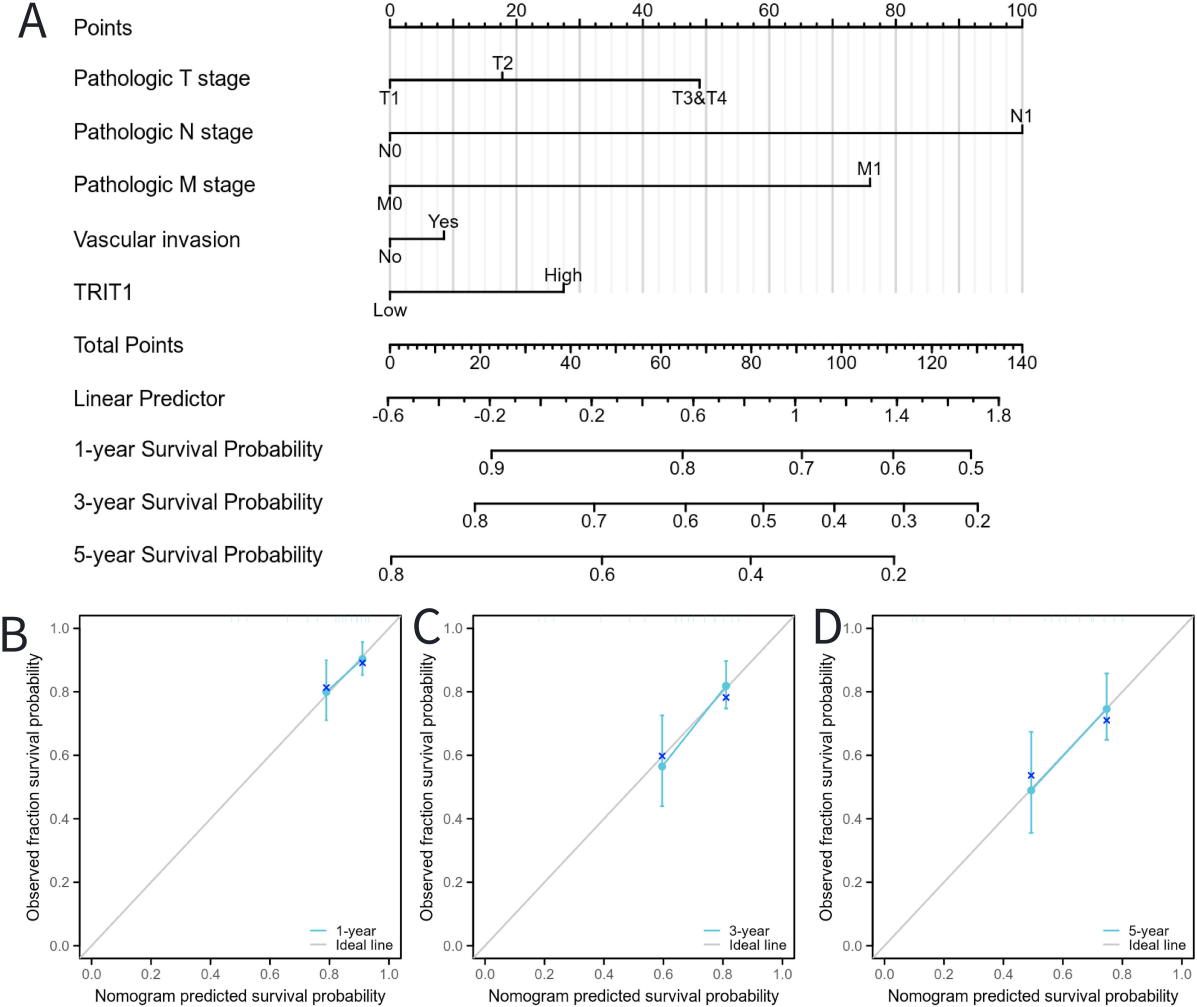
Calibration curves and a nomogram for predicting OS rates of LIHC patients. **(A)** A nomogram chart was a visual representation that displays the data of LIHC patients’ OS rates at specific time intervals, such as one, three, and five years. **(B–D)** Calibration curves were graphical tools used to predict the survival rates of cancer patients at specific time points.

To predict the prognosis of patients diagnosed with LIHC, an extensive analysis was conducted to evaluate multiple independent factors. This is essential as multiple factors can influence the prognosis of LIHC patients. Based on these factors, we developed a comprehensive nomogram. The developed nomogram demonstrated a significant inverse relationship between total risk scores and clinical outcomes in LIHC patients. Elevated nomogram scores, indicative of greater cumulative adverse prognostic factors, showed strong association with poorer survival outcomes (**Figure 8A**). This dose-response relationship suggests the nomogram’s clinical utility for risk stratification, where increasing point totals correspond to progressively worse prognosis. This relationship shows the practical significance of the nomogram in predicting patient outcomes. The predictive reliability of the nomogram was further assessed through comprehensive calibration curve analysis (**Figures 8B-D**). These validation analyses demonstrated consistent agreement between predicted and observed clinical outcomes across multiple time points. Through this systematic investigation, TRIT1 was identified as a significant LIHC-associated gene and independent prognostic factor. The findings establish TRIT1 as a molecular determinant influencing LIHC progression and patient outcomes.

## Discussion

Liver hepatocellular carcinoma (LIHC) is one of the leading causes of cancer-related death worldwide. Its incidence continues to rise, especially in western countries (10). Moreover, its complex pathological mechanisms and heterogeneity pose great challenges to diagnosis and treatment. Currently, there is a lack of effective biomarkers to accurately predict the prognosis of liver cancer and guide personalized treatment. There have been a number of studies on comprehensive bioinformatics analysis to identify biomarkers or therapeutic targets related to the prognosis of hepatocellular carcinoma, providing new ideas and directions for the diagnosis, treatment and prognosis assessment of hepatocellular carcinoma. They have also proven the importance of bioinformatics methods in the discovery of prognostic markers of liver cancer (11, 12), offering additional support for the research. Although this study differs from others in the selection of prognostic markers, all studies highlight the complexity and heterogeneity of liver cancer, which provides new perspectives for the diagnosis and treatment of liver cancer. This investigation employed the TCGA database to systematically analyze TRIT1 expression patterns in hepatocellular carcinoma. The study was designed to explore potential applications of TRIT1 in early diagnostic strategies and prognostic evaluation for liver cancer.

TRIT1 exhibits pan-cancer overexpression, with significant upregulation in LIHC (**Figures 1A-C**). TRIT1 may affect cell homeostasis through the formation of amyloid fibers, thus promoting tumor progression (13). Phenotypes caused by defects in TRIT1, such as myoclonic epilepsy and delayed speech, may reflect its importance in maintaining cell homeostasis. This, in turn, may be associated with the development of liver cancer (14).

In addition, the high expression of TRIT1 in liver cancer may also be related to the abnormal metabolism of liver cancer cells. First, TRIT1 can catalyze the isopentenization of tRNA[Ser]Sec, which is related to the expression of selenoprotein (15). Selenoproteins play a crucial role in regulating cellular metabolism, including antioxidant stress, signaling, and protein synthesis (16, 17). Many enzymes in selenoproteins participate in intracellular energy metabolism processes, such as the tricarboxylic acid cycle (TCA cycle) and pentose phosphate pathway (PPP) (18). Therefore, it is reasonable to infer that TRIT1 may affect the energy metabolism of hepatoma cells by regulating the expression of selenoprotein, affecting their growth and survival ability. Moreover, selenoproteins also play a key role in regulating cellular metabolic pathways. Specifically, the high expression of TRIT1 may affect the metabolic pathways, such as glycolysis, lipid metabolism and amino acid metabolism, by affecting the expression and function of selenium protein (18).This further indicates the far - reaching impact of TRIT1 on the metabolic activities of liver cancer cells.

In addition to metabolism, selenoproteins play an important role in cell cycle regulation. By affecting the expression of selenium protein, TRIT1 may affect the proliferation and cell cycle process of liver cancer cells, which can not only promote the growth of normal cells, but also inhibit the proliferation of cancer cells, even inducing their apoptosis (19). This suggests that the high expression of TRIT1 might have a dual - edged effect on the growth of liver cancer cells through selenoproteins.

Furthermore, the high expression of TRIT1 may affect the drug sensitivity and resistance of cancer patients by influencing the expression and function of selenoprotein (20–22).

In conclusion, the high expression of TRIT1 in liver cancer may have a significant impact on various cellular processes, including metabolism, proliferation, energy production, and drug sensitivity, by modulating the expression and function of selenoproteins. Consequently, it may facilitate the progression of liver cancer. The findings offer novel understanding of TRIT1’s role in liver cancer and identify potential therapeutic targets for the treatment of this disease.

Following computational analysis of TCGA datasets, the differential expression of TRIT1 was further corroborated in clinical specimens. Experimental validation confirmed significantly differential TRIT1 expression between hepatocellular carcinoma (LIHC) and normal liver tissues (**Figure 2**). ROC curve analysis further demonstrated that TRIT1 gene expression exhibited high predictive accuracy for liver cancer, with an AUC value of 0.854 (**Figure 1D**). These findings suggest TRIT1 has the potential to be a diagnostic biomarker for liver cancer. Notably, TRIT1 is also an emerging candidate oncogene, as its high expression has been reported in small cell lung cancer (23). Therefore, TRIT1 expression could serve as a valuable addition to the existing panel of biomarkers for liver cancer diagnosis.

In this study, the methylation status of TRIT1 DNA sequence methylation sites was systematically analyzed in LIHC. The analysis revealed that certain methylation sites in the TRIT1 DNA sequence were hypermethylated in LIHC (**Figure 1E**). Hypermethylation at specific TRIT1 loci was hypothesized to regulate gene expression patterns, potentially leading to a poor prognosis in liver cancer. Previous comprehensive analyses of DNA methylation have shown that abnormal changes in DNA methylation across the entire genome play a significant role in the progression of liver cancer from normal liver tissue to early stages. These findings suggest a close relationship between overall DNA methylation patterns and the occurrence and development of liver cancer, providing a broader context and basis for studying the role of DNA sequence methylation in specific genes like TRIT1 in liver cancer (24).

The junctions in the DNA structure suggest that methylation might play a role in tumorigenesis and its progression. Changes in DNA methylation patterns are considered an early event in tumor development, which can influence the biological behavior of tumors by regulating gene expression (25). Studies have demonstrated that DNA methylation plays a pivotal role in regulating gene expression, especially in the development and progression of cancers. For instance, the interplay between DNA methylation, gene expression, and cellular free DNA fragmentation has been explored. Higher levels of circulating free DNA (cfDNA) and larger fragment sizes have been associated with reduced CpG methylation and gene expression (26). Additionally, in the context of DNA methylation, DNA methyltransferases hold a key position in tumorigenesis because their dysregulation often leads to the activation of oncogenes and the inactivation of tumor suppressor genes (27). These discoveries emphasize the significance of early events related to DNA methylation patterns in tumorigenesis and the crucial role of gene expression regulation. They offer a fresh perspective for further exploring the biological behavior of tumors. Not only does the pattern of DNA methylation in cancer affect diagnosis and prognosis, but it also has crucial implications for treatment and the development of novel therapeutic strategies (28).

However, it is important to note that the correlation between the DNA methylation level of TRIT1 and the prognosis of liver cancer lacks statistical significance, and the underlying mechanism remains incompletely understood. Future investigations should delve into the precise manner in which TRIT1 methylation influences its expression and how this influence manifests in the clinical presentation of liver cancer. Furthermore, considering the dynamic shifts in methylation levels, further exploration is warranted to assess TRIT1’s role in different stages of liver cancer and its potential integration into clinical decision-making processes.

This study additionally revealed a correlation between elevated TRIT1 expression and unfavorable clinicopathological markers of liver cancer. Specifically, higher levels of TRIT1 were associated with larger tumor sizes (T3) and advanced disease stages (stage III) (**Figures 3A-E**). These observations provide further evidence for TRIT1’s potential role in promoting liver cancer progression. Given TRIT1’s subcellular specificity, it could serve as a potential therapeutic target for liver cancer. The development of treatment strategies that specifically target TRIT1’s mitochondrial and cytoplasmic functions could offer novel avenues for liver cancer management (29).

The association between TRIT1 expression and LIHC prognosis was evaluated using Kaplan-Meier survival analysis. This analytical approach enabled comprehensive evaluation of TRIT1’s prognostic impact across distinct LIHC patient subgroups. Existing prognostic studies corroborate these findings, demonstrating consistent associations between TRIT1 expression and LIHC outcomes (30). High TRIT1 expression levels demonstrated significant association with adverse prognosis in multiple LIHC subpopulations, suggesting potential pan-subgroup oncogenic effects. In recent years, numerous studies have emphasized the profound impact of the tumor microenvironment on the progression of tumors, particularly in the context of liver cancer (31–34). Experimental evidence reveals that tumor-stromal crosstalk, particularly through fibroblast- and immune cell-derived factors, critically drives tumor cell proliferation (35). Specifically, in terms of immune cell infiltration, the current findings indicate a potential correlation between TRIT1 expression and the degree of infiltration by immune cells, specifically pDCs, Cytotoxic cells, and B cells (**Figure 7A**). This correlation may provide insight into the response to immunotherapy in liver cancer. The complexity and heterogeneity of the immune microenvironment in liver cancer offer novel opportunities for immunotherapy strategies (36). Given TRIT1’s role in the immune microenvironment of liver cancer, it emerges as a potential target for immunotherapy. This finding underscores TRIT1’s crucial role in regulating the immune microenvironment in liver cancer. Some studies have categorized the immune microenvironment of liver cancer into three immune subtypes: high - immune, neutral - immune, and low - immune (37). TRIT1 provides a fresh perspective for evaluating this complex microenvironment. Moreover, in the management of liver cancer, understanding the immune microenvironment is paramount in developing effective treatment strategies (38). The significance of the liver cancer immune microenvironment in treatment underscores the need for further exploration and understanding of TRIT1’s role in this context. Overall, these findings contribute to a deeper understanding of the intricate interplay between the tumor and its microenvironment, particularly in the context of liver cancer immunotherapy.

Further analysis indicated that, compared to the low TRIT1 expression group, the enrichment fractions of B cells, Cytotoxic cells, and pDCs in the high TRIT1 expression group were significantly lower (**Figures 7B-H**), while the enrichment fraction of Tcm was significantly higher (**Figure 7I**). This suggests that TRIT1 may influence the immune microenvironment of liver cancer by inhibiting the infiltration of certain immune cells while promoting the accumulation of others. This finding aligns with existing literature discussing the heterogeneity of the liver cancer immune microenvironment, which emphasizes the diversity of immune cell types and their roles in tumor progression. Specifically, variations in the type, density, and spatial distribution of immune cells may significantly impact the prognosis of liver cancer. Moreover, the relationship between TRIT1 expression levels and the number of infiltrated immune cells further supports this observation (39). Additionally, relevant studies have established prognostic models for liver cancer patients, reinforcing the findings and highlighting the importance of understanding TRIT1’s role in the immune microenvironment (40).

Although this study offers a novel perspective on the correlation between TRIT1 levels and prognosis in patients with liver cancer (LIHC), several notable limitations exist that could potentially affect the reliability and generalizability of the findings. In terms of the diagnosis - related results, the retrospective nature of this study may lead to selection bias. Since retrospective studies rely on existing medical records, these records may contain missing or incorrect information. If the missing data are exactly related to the expression of TRIT1 and the prognosis of patients, it may lead to an underestimation or overestimation of the degree of the association between TRIT1 and prognosis. To comprehensively assess the role of TRIT1 in LIHC diagnosis, prognostic evaluation, and immunotherapy, future studies should aim for prospective validation of TRIT1. Secondly, the sample size of this study is relatively small, which could potentially affect the reliability of the statistical analysis results. A larger cohort would greatly enhance the study’s validity and reliability. While this study establishes TRIT1’s association with immune infiltration in LIHC at the transcriptional level, the mechanistic underpinnings—including upstream regulators and downstream effectors of TRIT1—remain to be elucidated. This gap currently precludes a comprehensive understanding of TRIT1-mediated immunomodulation in the tumor microenvironment. Additionally, this study does not discuss the potential application of TRIT1 in the treatment of LIHC patients. Future studies could aim to integrate TRIT1 into clinical treatment plans and analyze how TRIT1 expression levels may impact treatment outcomes. In summary, while this study provides valuable insights, further research is warranted to comprehensively understand the role of TRIT1 in LIHC diagnosis, prognosis, and treatment.

## Data Availability

The data presented in the study are deposited in the TCGA repository. The link is https://portal.gdc.cancer.gov/projects/TCGA-LUAD.
