# Supplementary material for: To determine the role of TRIT1 in the diagnosis, prognosis and immunoinvasion of liver hepatocellular carcinoma
Source: Front Immunol. 2025 May 5;16:1563442. doi: 10.3389/fimmu.2025.1563442 (PMC12086158; doi:10.3389/fimmu.2025.1563442)
Supplement: Supplementary file 1 [file Table1.docx]

**To Determine the Role of TRIT1 in the Diagnosis, Prognosis and Immunoinvasion of Liver Hepatocellular Carcinoma**

Xinyu Niu*^b^*, Xiaona Pan*^c^*, Guifang He*^d^*, Chao Xuan*^a^*, Qingwu Tian*^a^*, Yuan Yuan*^a^*, Jingqiu Chen*^b^*, Yaqi Song*^b^*, Yujuan Tang^e,f*2^, Tingting Zhou*^a^*^*1^

*^a^ Department of Clinical Laboratory,* *The Affiliated Hospital of Qingdao University, Qingdao 266003, Shandong, China*

*^b^ Qingdao University, Qingdao 266000, Shandong, China*

*^c^ Department of Rehabilitation Medicine, The Affiliated Hospital of Qingdao University, Qingdao 266003, Shandong, China*

*^d^ Medical Animal Laboratory, The Affiliated Hospital of Qingdao University, Qingdao 266003, Shandong, China*

*^e^ Department of Surgery, Hubei Provincial Hospital of Traditional Chinese Medicine,Wuhan 430061, Hubei, China*

*^f^ HubeiProvincial Hospital of Traditional Chinese Medicine, Affiliated Hospital of Hubei University of Chinese Medicine,Wuhan 430061, Hubei, China*

**Supplementary materials:**

**Table S1 Functional enrichment analyses**

| **ONTOLOGY** | **ID** | **Description** |
| --- | --- | --- |
| BP | GO:0006882 | intracellular zinc ion homeostasis |
| BP | GO:0046688 | response to copper ion |
| BP | GO:0055069 | obsolete zinc ion homeostasis |
| BP | GO:0046686 | response to cadmium ion |
| CC | GO:0031838 | haptoglobin-hemoglobin complex |
| CC | GO:0005833 | hemoglobin complex |
| CC | GO:0034385 | triglyceride-rich plasma lipoprotein particle |
| CC | GO:0042627 | chylomicron |
| MF | GO:0031720 | haptoglobin binding |
| MF | GO:0031210 | phosphatidylcholine binding |
| MF | GO:0005344 | oxygen carrier activity |
| MF | GO:0019825 | oxygen binding |
| KEGG | hsa05033 | Nicotine addiction |
| KEGG | hsa04972 | Pancreatic secretion |
| KEGG | hsa04976 | Bile secretion |
